# Optical coherence tomography angiography reveals progressive worsening of retinal vascular geometry in diabetic retinopathy and improved geometry after panretinal photocoagulation

**DOI:** 10.1371/journal.pone.0226629

**Published:** 2019-12-30

**Authors:** Alaa E. Fayed, Ahmed M. Abdelbaki, Omar M. El Zawahry, Amani A. Fawzi

**Affiliations:** 1 Department of Ophthalmology, Feinberg School of Medicine, Northwestern University, Chicago, Illinois, United States of America; 2 Department of Ophthalmology, Kasr Al-Ainy School of Medicine, Cairo University, Cairo, Egypt; Massachusetts Eye & Ear Infirmary, Harvard Medical School, UNITED STATES

## Abstract

**Purpose:**

To quantify vessel tortuosity and fractal dimension of the superficial capillary plexus (SCP) of the macula in different stages of diabetic retinopathy (DR), and following panretinal photocoagulation (PRP) using optical coherence tomography angiography (OCTA).

**Methods:**

75 eyes of 75 subjects were divided into five groups; healthy controls, diabetes with no clinical DR, non-proliferative diabetic retinopathy (NPDR), proliferative diabetic retinopathy (PDR) and patients who received PRP for PDR (PDR+PRP).For vessel tortuosity, SCP slabs from 3x3 mm macular OCTA scans were processed using imageJ (NIH, USA), where large perifoveal vessels were traced and their length was measured with tortuosity calculated as the ratio between the actual length and the straight Euclidean length. For fractal dimension, SCP slabs were processed and imported to Fractalyse (Th*é*MA, France), where box-counting analyses produced fractal dimension values.

**Results:**

We found a significant difference in vessel tortuosity and fractal dimension between the five groups (one-way ANOVA, p < 0.001both). NPDR and PDR had significantly more tortuous vessels and lower fractal dimension compared to healthy controls (Tukey HSD: p = 0.02, 0.015,0.015 and <0.001, respectively). Fractal dimension was also significantly lower in NPDR and PDR compared to eyes with no clinical DR (p <0.001 both), and in PDR compared to NPDR (p = 0.014). Following PRP, vessel tortuosity was significantly lower and fractal dimension was higher in PDR+PRP compared to PDR (p = 0.001 and 0.031, respectively).

**Conclusions:**

We used macular OCTA scans to demonstrate significantly higher perifoveal large vessel tortuosity, and lower fractal dimension in NPDR and PDR compared to healthy controls. Vessel tortuosity shows more dramatic normalization than fractal dimension and could be explored as a sensitive marker for successful PRP.

## Introduction

Retinal vessel tortuosity and retinal vascular fractal dimension are global measures of retinal vascular geometry that have been studied intensely.[1–3] Increased vessel tortuosity is thought to reflect an increase in the hydrostatic pressure as a response to autoregulatory arteriolar dilatation caused by tissue hypoxia.[4] This can be explained by Laplace’s law which suggests that in response to high hydrostatic pressure, there would be an increase in vessel diameter. Further, according to Young’s modulus, an increase in vessel length, i.e. tortuosity, eventually occurs when the pressure increase is large enough to overcome vessel wall stiffness.[5, 6]

The impact of diabetes on retinal vessel tortuosity has been studied extensively in literature.[1, 7–9] Higher HbA1c has been associated with increased arteriolar tortuosity in type 1 diabetic patients with non-proliferative diabetic retinopathy.[1] Furthermore, greater arteriolar tortuosity has been associated with a higher incidence of diabetic retinopathy (DR), as well as grade 1 nephropathy.[9–12] Large retinal vessels were more tortuous in diabetic eyes compared to healthy controls.[7, 13] One study went as far as to suggest that increased vessel tortuosity and elongation may predict the development of diabetic macular edema (DME).[5]

Fractal dimension, on the other hand, describes the degree of "complexity" of the branching retinal vascular network.[3, 14] Since DR is a microangiopathy known to cause capillary loss, it is logical that the branching architecture and level of complexity of the retinal vasculature should be altered.[15] Fractal dimension has been found to be lower, i.e.: less complex, in DR than in healthy controls.[16, 17]

These previous studies have largely focused on large vessels at the optic disc using either color fundus photography or fluorescein angiography.[11, 18, 19] Therefore, these results may not accurately reflect the perifoveal vasculature in DR with their impact on the integrity of the fovea and central vision. More recent studies have used optical coherence tomography angiography (OCTA) to study the geometry of the macular microvascular network in DR.[20–22] Lee et al[22] reported an increasing trend in vessel tortuosity in both the superficial and deep capillary plexuses (SCP and DCP) with increasing severity of DR. On the other hand, fractal dimension was found to be significantly lower in the SCP and DCP of eyes with non-proliferative diabetic retinopathy than healthy controls.[20, 23] These studies did not clearly focus on the impact of the different stages of DR on vascular geometry, as well as the effect, if any, of PRP. Another limitation of these studies was the inclusion of the small perifoveal capillaries in image analysis software modules. Although changes in vessel tortuosity have been documented in capillaries in DR,[13] these smaller capillaries are extremely susceptible to various confounding factors, such as image quality, capillary dropout secondary to non-perfusion and the presence of DME.[24–26]

In this study, we used 3x3 mm OCTA macular scans of the SCP to study large first and second order macular vessel tortuosity and overall fractal dimension. We compared the findings in five disease severity groups: healthy controls, DM with no clinical DR, non-proliferative diabetic retinopathy (NPDR), treatment naïve proliferative diabetic retinopathy (PDR) and quiescent PDR eyes following panretinal photocoagulation for high-risk PDR (PDR+PRP). To address the limitations in previous studies, we applied strict patient inclusion criteria and focused on the perifoveal arterioles and venules in the SCP to avoid the potential confounding effects of the smaller capillaries on vessel tortuosity. We hypothesized that vessel tortuosity would have a positive correlation, while fractal dimension would have a negative correlation with increasing severity of DR. We also hypothesized that PRP may reverse these parameters to reflect the improvement in the vascular integrity and perfusion of the posterior pole.[27]

## Patients and methods

This was a retrospective analysis of healthy participants and patients with diabetes recruited in the Department of Ophthalmology at Northwestern University in Chicago, Illinois between June 2015 and January 2019. The study was approved by the institutional review board of Northwestern University, followed the tenets of the Declaration of Helsinki, and was performed in accordance with Health Insurance Portability and Accountability Act regulations. Written informed consent was obtained from all participants.

### Study sample

Inclusion criteria were healthy eyes, eyes from patients with diabetes mellitus (DM) without DR, eyes with NPDR, or eyes with PDR based on clinical assessment by a retina specialist (A.A.F).For healthy controls, we excluded subjects if they had a history of DM, systemic hypertension (HTN) or cardiovascular disease (CVD) or were known current or past smokers. For the DM with no clinical DR group, we excluded patients with history of HTN or CVD or were known current or past smokers. For NPDR and PDR patients, we included treatment-naïve eyes with a fundus photograph obtained within one month of the OCTA scan. The fundus photograph was used to diagnose and include moderate and severe NPDR, as well as high-risk PDR based on the ETDRS classification.[28] We only included moderate and severe NPDR, but not mild and very mild NPDR, to effectively reflect the microvascular changes in NPDR, and avoid possible bias or overreach in diagnosing cases with no clinically visible DR as very mild NPDR. Moderate NPDR was diagnosed as having at least one of the following fundus findings: severe retinal hemorrhages (more than ETDRS standard photograph 2A: about 20 medium–large per quadrant) in 1–3 quadrants, mild IRMA, significant venous beading in no more than 1 quadrant or cotton wool spots. Severe NPDR was diagnosed using the 4-2-1 rule by having at least one of the following: severe hemorrhages in all 4 quadrants, significant venous beading in 2 or more quadrants or moderate IRMA in 1 or more quadrants. High-risk PDR was diagnosed by having at least one of the following: new vessels on the disc (NVD) greater than ETDRS standard photograph 10A (about a third of the disc area), any NVD with vitreous hemorrhage or new vessels elsewhere (NVE) greater than half the disc area with vitreous hemorrhage. For PDR+PRP patients, we only included those who have been stable (no recent development of neovascularization) for at least 1 year and have not received any other form of ocular treatment. The rationale behind the 1-year interval was to ensure the stability and tight control of the proliferative element of DR, and determine whether the vascular changes seen after PRP were long-lasting. For all patients, only eyes that had OCTA images without significant movement or shadow artifacts, a quality index (Q) of 6 or more and a signal strength index (SSI) score above 50 were considered eligible.

Exclusion criteria were eyes with other retinal or choroidal diseases that may confound our results, eyes with center-involving DME and eyes that have received intravitreal anti-VEGF agents, intravitreal triamcinolone acetonide, dexamethasone intravitreal implant, focal laser or pars plana vitrectomy. We also excluded eyes with astigmatism (more than 3 diopters), high refractive error (more than 6 diopters), or cataract graded above nuclear opalescence grade three or nuclear color grade three, to avoid optical artifacts that potentially may compromise OCTA image quality. Electronic medical records were reviewed to extract demographic and clinical information.

### OCT Angiographic imaging and image processing

Patients underwent imaging using RTVue-XR Avanti device (Optovue Inc, Fremont, California, USA), with split-spectrum amplitude-decorrelation angiography (SSADA) software.[29] This instrument has an A-scan rate of 70,000 scans per second and uses a light source centered at 840 nm and a bandwidth of 45nm. Two consecutive B-scans (M-B frames), each containing 304 A-scans, were captured at each sampling location and SSADA was used to extract OCTA information. 3D Projection artifact removal (3D-PAR) technology by Optovue was used to obtain 3x3 mm scans centered on the fovea.[30] *En face* OCT angiograms were segmented automatically using the built-in software to define the SCP. The inner boundary of the *en face* image segment was set at the internal limiting membrane (ILM), and the outer boundary was set at 10 μm above the inner plexiform layer (IPL).

To eliminate the risk of bias, the images were randomized and the grader (A.E.F) was masked to the randomization process. The measurements were done twice (by the same grader) to ensure the validity and repeatability of the results, and repeatability was evaluated by statistics, as explained below.

### Vessel tortuosity calculation

**Fig 1:**
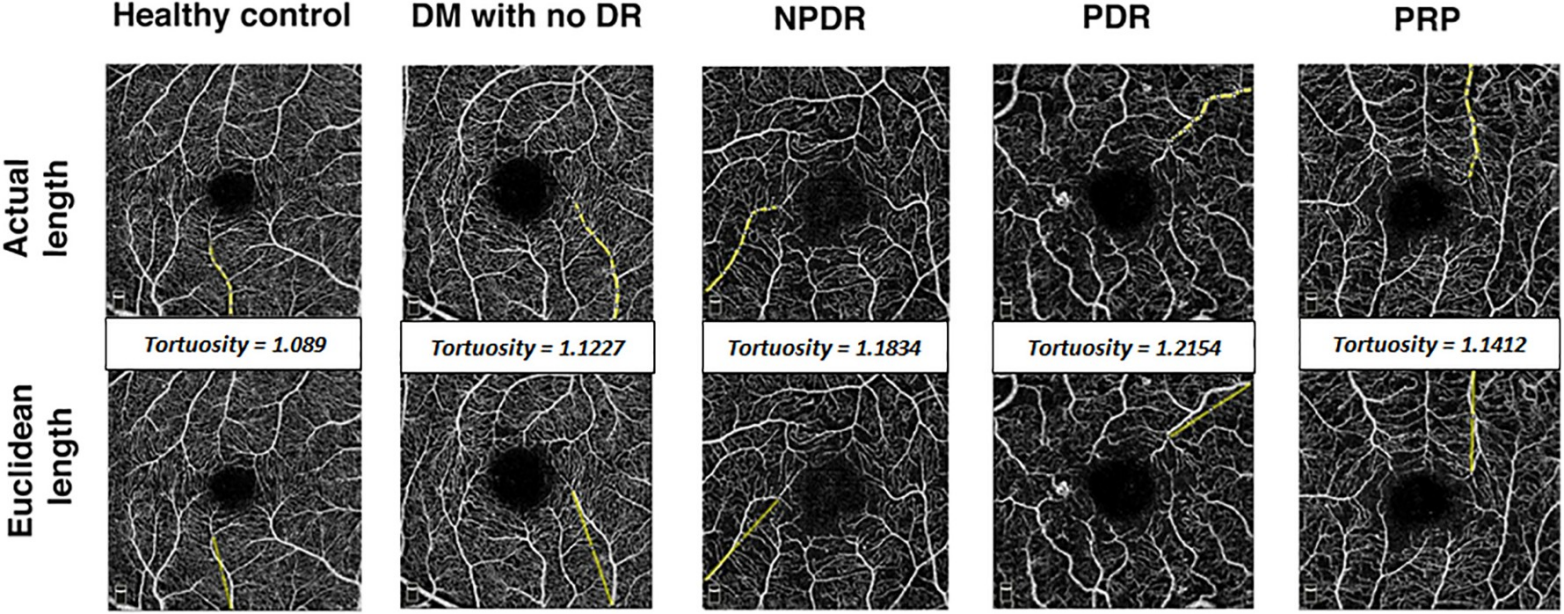
Calculation of vessel tortuosity. 3x3 mm OCTA scans segmented to show the SCP (inner boundary at the ILM and the outer boundary 10 μm above the IPL). The actual and Euclidean lengths are measured for every individual branch and vessel tortuosity is calculated as the ratio between the sum of the actual lengths of all branches and the sum of their Euclidean distances.

SCP images were imported into ImageJ software (National Institutes of Health [NIH], Bethesda, Maryland, USA).[31] We determined all large first and second order perifoveal arterioles and venules, traced them and their length was measured using 2 different methods. First, we traced the actual path of the vessel segment from its origin, either at the edge of the OCTA scan or the branch-point origin, until its final branching point at the foveal avascular zone. This was recorded as the "actual length" of the vessel. Then we measured the length of a straight line starting from the same origin point, and extending directly to the end point. This was recorded as the "Euclidean distance length". Vessel tortuosity was estimated as the ratio between the sum of the actual length of all the vessels and the sum of their Euclidean distance length (Fig 1).[32, 33] Retinal tortousity, quantified using the Euclidean distance, has been used to study peripapillary and bulbar conjunctival vessel tortuosity in diabetic retinopathy[7, 34], correlation between retinal vascular tortuosity in diabetic retinopathy and renal disease[11], sickle cell retinopathy[35], characterization of familial retinal arteriolar tortuosity [36], and diagnosis and quantification of plus disease in retinopathy of prematurity.[37, 38]

### Fractal dimension calculation

SCP images were imported and binarized using ImageJ.[31] Fractal dimensional box-counting analyses were performed using Fractalyse (ThéMA, Besançon Cedex, France) resulting in a fractal dimension value for each image.[39] In this analysis, a count is made of the number of squares subtended by a pattern in an image; this count is repeated as the size of the squares of the grid is reduced. This method is used to compare the degree of complexity among different vascular patterns. The box-counting fractal dimension is given by the relationship:

$$Ds=\frac{\text{log}\;Nr}{\text{log}\;r-1}$$

where D_s_ is the fractal dimension as measured by box counting and N_r_ is the number of boxes subtended by boxes of ratio r.[40]

### Statistical analysis

We used IBM SPSS statistics version 25 (IBM SPSS Statistics; IBM Corporation, Chicago, IL, USA). Intra-class correlation coefficient (ICC) for the repeatability of the measurement by the single grader (intra-grader repeatability) was 0.927 and 1.0 for vessel tortuosity and fractal dimension, respectively. Shapiro-Wilk tests were used to determine if data was distributed normally. The difference between sexes within each of the five groups was determined by the chi-square test. One-way analysis of variance (ANOVA) was used to examine the global difference between the five groups in terms of age, duration of DM and HbA1c, as well as vessel tortuosity and fractal dimension. Post-hoc Tukey Honest Significant Difference (HSD) analyses were run between group pairs. A p value of <0.05 was considered statistically significant.

## Results

The study included 15 eyes of 15 patients in each of the five study groups: healthy control, DM with no clinical DR, NPDR, PDR and PDR+PRP, for a total of 75 eyes of 75 patients. The overall demographics and disease-related characteristics are reported in Table 1. There was no statistically significant difference between the five groups in age, sex and HbA1c. There was, however, a statistically significant difference in duration of DM between the five groups (one-way ANOVA, p < 0.001).

**Table 1:** Demographic and disease-related patient characteristics.

	*Healthy controls*	*DM with no DR*	*NPDR*	*PDR*	*PRP*	*p-value*
**Patients (n)**	15	15	15	15	15	
**Sex** **Female, n (%)**	6 (40)	8 (53.3)	8 (53.3)	4 (26.7)	5 (33.3)	0.475
**Male, n(%)**	9 (60)	7 (46.7)	7 (46.7)	11 (73.3)	10 (66.7)	
**Age; years** **(mean ± SD)**	53 ± 14	51 ± 9	50 ± 12	44 ± 10	53 ± 15	0.182
**Disease duration; years** **(mean ± SD)**	N/A	8 ± 5	13 ± 6	16 ± 8	24 ± 11	**< 0.001**
**HbA1c; %** **(mean± SD)**	N/A	8.22 ± 1.73	9.01 ± 2.14	9.42 ± 2.06	8.42 ± 1.71	0.308

DM with no DR: Diabetes mellitus with no diabetic retinopathy, NPDR: Non-proliferative diabetic retinopathy, PDR: Proliferative diabetic retinopathy, PRP: Panretinal photocoagulation, HbA1c: Hemoglobin A1c, p-value based on one-way ANOVA comparison between groups.

### Vessel tortuosity

**Fig 2:**
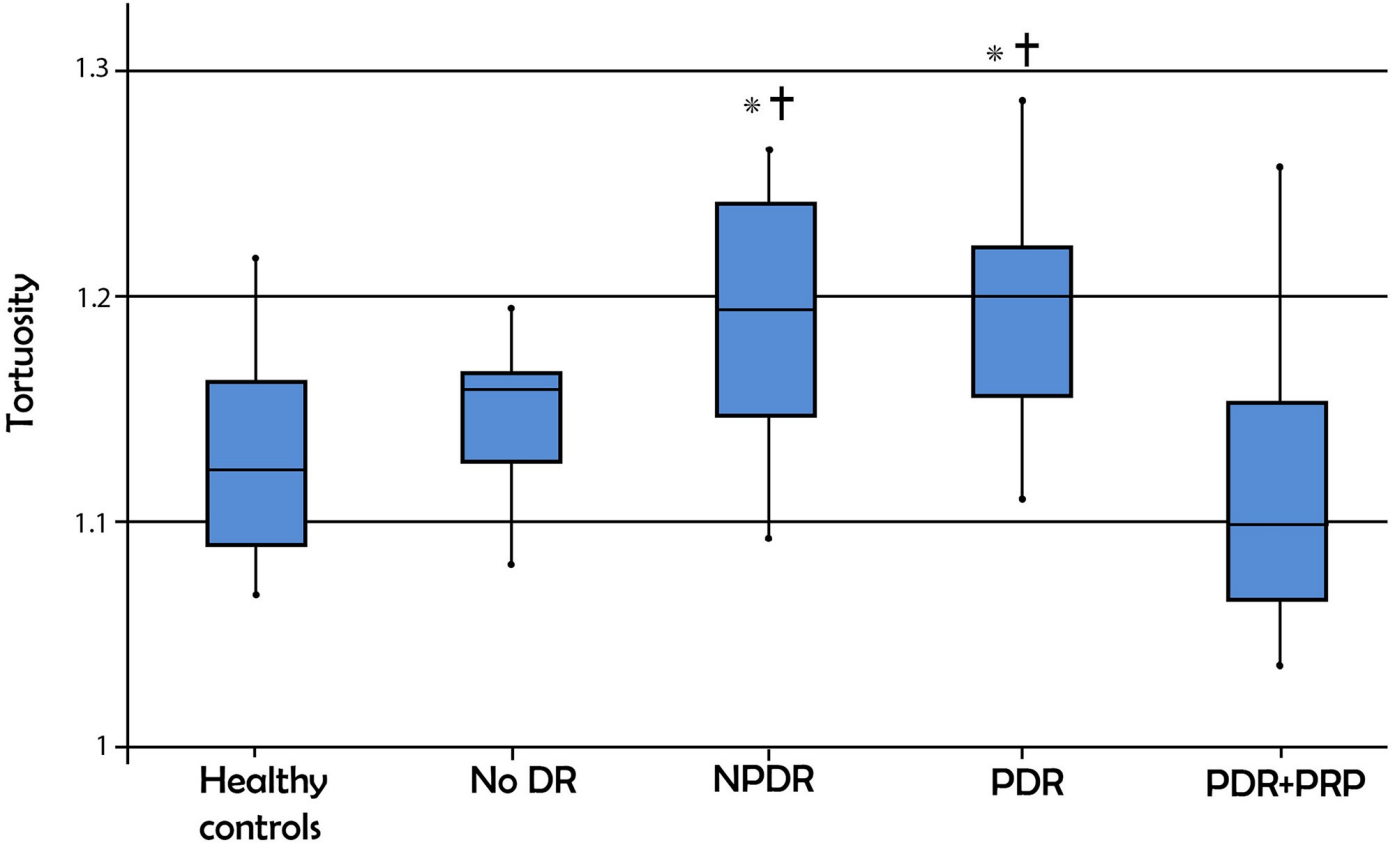
Box plot representation of the perifoveal vessel tortuosity results in the five study groups. No DR: No diabetic retinopathy, NPDR: Non-proliferative diabetic retinopathy, PDR: Proliferative diabetic retinopathy, PRP: Panretinal photocoagulation, PDR+PRP: Proliferative diabetic retinopathy receiving panretinal photocoagulation. (p<0.05,*significant vs Healthy controls, † significant vs PDR+PRP).

One-way ANOVA showed a significant difference in vessel tortuosity between the five groups (p < 0.001; Fig 2). The NPDR and PDR groups had significantly more tortuous vessels compared to the healthy control group (Tukey HSD: p = 0.02 and 0.015, respectively). Following PRP, vessel tortuosity was significantly lower in the PDR+PRP group when compared to either the NPDR or PDR groups (Tukey HSD p = 0.001). The remaining results were not significant and are outlined in Table 2.

**Table 2:** Post-hoc analysis of vessel tortuosity and fractal dimension results comparing the five groups.

	*Pair-wise comparison*	*Tortuosity p value*	*Fractal dimension p value*
**Healthy controls**	*No DR*	0.947	0.820
	*NPDR*	**0.02**	**0.015**
	*PDR*	**0.015**	**< 0.001**
	*PRP*	0.909	**0.006**
**DM with no DR**	*Healthy controls*	0.947	0.820
	*NPDR*	0.126	**< 0.001**
	*PDR*	0.102	**< 0.001**
	*PRP*	0.502	**< 0.001**
**NPDR**	*Healthy controls*	**0.02**	**0.015**
	*No DR*	0.126	**< 0.001**
	*PDR*	0.98	**0.014**
	*PRP*	**0.001**	0.999
**PDR**	*Healthy controls*	**0.015**	**< 0.001**
	*No DR*	0.102	**< 0.001**
	*NPDR*	0.98	**0.014**
	*PRP*	**0.001**	0.031
**PRP**	*Healthy controls*	0.909	**0.006**
	*No DR*	0.502	**< 0.001**
	*NPDR*	**0.001**	0.999
	*PDR*	**0.001**	**0.031**

No DR: No diabetic retinopathy, NPDR: Non-proliferative diabetic retinopathy, PDR: Proliferative diabetic retinopathy, PRP: Panretinal photocoagulation, statistically significant Tukey HSD:p< 0.05.

### Fractal dimension

**Fig 3:**
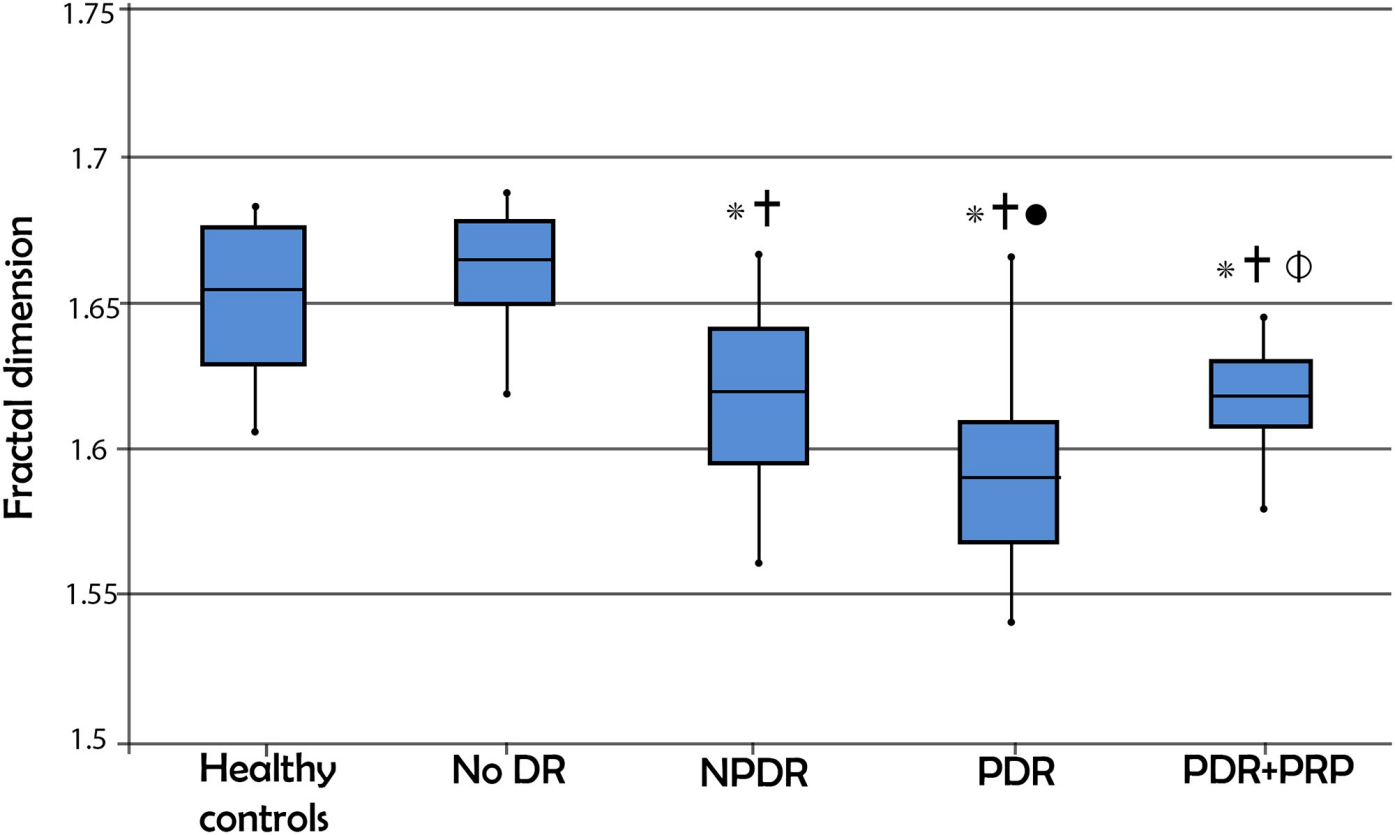
Box plot representation of the fractal dimension results in the 5 study groups. No DR: No diabetic retinopathy, NPDR: Non-proliferative diabetic retinopathy, PDR: Proliferative diabetic retinopathy, PRP: Panretinal photocoagulation, PDR+PRP: Proliferative diabetic retinopathy receiving panretinal photocoagulation. (p<0.05,*significant vs Healthy controls,†significant vs no clinical DR, ● = significant vs NPDR, ɸ significant vs PDR).

Fig 3 demonstrates the fractal dimension results for the five groups. One-way ANOVA showed a significant difference in fractal dimension when comparing the five groups (p < 0.001). Fractal dimension was significantly lower in the NPDR and PDR groups compared to the healthy control group (Tukey HSD: p = 0.015 and <0.001, respectively) or to the DM with no DR group (Tukey HSD: p <0.001 for both). Fractal dimension was also significantly lower in the PDR group compared to the NPDR group (Tukey HSD: p = 0.014). Fractal dimension in the PDR+PRP group was significantly higher than the PDR group (Tukey HSD p = 0.031), and significantly lower than the healthy controls (Tukey HSD p = 0.006). The remaining results were not significant and are outlined in Table 2.

## Discussion

In this study we evaluated the correlation between the superficial perifoveal vascular geometry and DR severity in untreated eyes and those that have received PRP. We found statistically significant higher tortuosity and lower fractal dimension with increasing severity of DR (p< 0.001 for both). None of the parameters were different when comparing healthy eyes and those with DM without DR. In the DM without DR group, the perifoveal fractal dimension was significantly higher than the NPDR, while tortuosity was not different. In NPDR and PDR, tortuosity was significantly higher than healthy controls, and fractal dimension was significantly lower. When comparing NPDR to PDR, fractal dimension was significantly lower in PDR, while tortuosity was not significantly different. From these results we can deduce that fractal dimension performs better at discriminating the stages of DR: no DR vs NPDR vs PDR, than perifoveal tortuosity. Quiescent PDR eyes after PRP show significantly lower perifoveal vessel tortuosity and higher fractal dimension than untreated PDR eyes, suggesting that both parameters are able to capture the improved macular hemodynamic status after PRP.

Various studies have made the connection between alterations in retinal vascular geometry and early stages of DR.[41–46] Fractal dimension of the peripapillary and perifoveal vessels has been found to be significantly lower in NPDR compared to healthy control eyes or eyes without clinical DR.[16, 17, 20, 23] Our results agree with these findings and confirm that the onset of clinical DR has a global impact on retinal vascular fractal dimension. Studies of peripapillary vessel tortuosity reported significantly more tortuous arterioles in NPDR eyes compared to healthy controls, which is also consistent with our findings in perifoveal vessels, though we did not examine arterioles and venules separately. In contrast to our study, researchers studying peripapillary vasculature found significantly more tortuous arterioles in eyes without clinical DR compared to healthy eyes, and in eyes with NPDR compared to those without clinical DR.[7, 9, 47] We did not find significantly more tortuous macular vessels in these pair-wise comparisons, which is consistent with another study of macular OCTA.[22] This discrepancy can be explained by understanding the effect of the increased hydrostatic pressure associated with DR on different sized vessels. A model demonstrated by Kylstra et al[6] proposes that a critical level of transmural pressure must be reached before tortuosity becomes evident. It is thus plausible that the smaller perifoveal vessels are more resilient and experience this critical pressure in more advanced NPDR and PDR stages compared to the larger, first order, peripapillary vasculature.[48]

In PDR, our study shows significantly lower fractal dimension compared to either NPDR or healthy control eyes. This is in agreement with Ashraf et al[49], who also reported statistically significant lower fractal dimension in eyes with PDR than those with NPDR in both the SCP and DCP. This suggests that fractal dimension may be a consistently sensitive marker to the higher levels of microvascular ischemia in PDR, and is able to reflect the global impact of this ischemia on various retinal capillary plexuses. Comparing fractal dimension to perifoveal tortuosity, PDR patients showed significantly more tortuous vessels compared to healthy controls but not compared to NPDR. Contrary to our results, Lee et al [22] noted a significant decrease in tortuosity in PDR compared to NPDR patients. The reason for this discrepancy can be construed from differences in approach since they measured global tortuosity, including capillaries. As reported in their results, some of their patients showed large areas of capillary dropout, which could have artifactually lowered their overall tortuosity. This is one of the main limitations of their approach, which we overcame by specifically tracing the larger arterioles and venules to avoid the confounding effects of these capillary dropouts. Also in contrast to our results, Rasmussen et al [33] and Sasongko et al[7] did not document significantly more tortuous peripapillary vessels in eyes with PDR compared to healthy controls. One possible explanation for this discrepancy relates to study population differences. These prior studies combined treated and untreated PDR patients, while we studied these groups separately.

PRP has been established as a highly effective tool for high-risk PDR.[50] The mechanism has been debated, but animal studies have shown that PRP causes an increase in the partial pressure of oxygen (PO_2_) in the retina,[51, 52] which may be the underlying basis for the overall reduction in total retinal blood flow in response to the improved oxygenation.[53, 54] This overall decreased flow is more prominent in the retinal periphery and is counterbalanced by improved macular perfusion, as recently documented on OCTA by our group.[27] Higher tortuosity is thought to be a marker of tissue hypoxia and vascular endothelial growth factor (VEGF) production.[55, 56] Torp et al [57] demonstrated a decrease in peripapillary vessel tortuosity 6 months following successful PRP (defined as no further development of neovascularization or decline in visual acuity compared to baseline). We propose that the significant decrease in perifoveal tortuosity one year following successful PRP in the current study is in line with improved macular perfusion on OCTA [27], which would explain the decline in vessel tortuosity.

Similar to the trend detected in tortuosity, fractal dimension was significantly higher in the PDR+PRP eyes compared to those with untreated PDR. However, unlike tortuosity which was no longer significantly different from healthy controls after PRP, fractal dimension remained significantly lower. This demonstrates a more dramatic normalization of vessel tortuosity in PDR eyes treated with PRP, compared to fractal dimension and suggests that perifoveal vascular tortuosity can perhaps be explored as a sensitive marker for successful PRP in future longitudinal studies (Figs 2 and 3).

Not only do these findings further our understanding of the microvascular implications of DR, and confirm the global nature of these changes, but they also suggest that some of these implications may be slightly reversible. As documented by our study, the ability of laser treatment to alter and reverse the geometric architecture of the perifoveal vasculature suggests that these parameters may be incorporated into other longitudinal studies, and extrapolated to various imaging tools, to determine the possibility of providing prognostic insight, or predicting eyes with PDR that may require additional laser treatment, adjuvant therapy or closer follow up.

The strengths of this study include the strict patient selection criteria and the study of a population of PDR+PRP patients. Limitations include the retrospective nature and the relatively small sample size. Moreover, OCTA operates based on a specific threshold, which implies that smaller vessels with flow that is lower than that threshold may be missed. The impact of this limitation is minimized by discarding poor quality images or those with extensive motion artifact. Another limitation is the cross-sectional nature, which did not allow us to examine the effect of PRP in the same population of PDR subjects.

In conclusion, we used 3x3 mm macular OCTA scans of the SCP to demonstrate that vessel tortuosity is significantly higher, while fractal dimension is significantly lower in NPDR and PDR compared to healthy controls. Fractal dimension can be potentially used to screen and follow patients with no clinical DR as it changes significantly with the onset of clinical DR. PDR has a more significant impact on fractal dimension than vessel tortuosity, while PRP results in more significant normalization of tortuosity than fractal dimension. Therefore, we suggest that tortuosity could perhaps be explored as a sensitive marker for successful PRP. Overall our results provide novel insights that could have potentially important clinical applications.

## Supporting information

S1 Dataset Excel sheet describing the perifoveal tortuousity and fractal dimension of each one of the 75 eyes in the five studied groups: Healthy controls, diabetics with no clinical diabetic retinopathy, non-proliferative diabetic retinopathy (NPDR), proliferative diabetic retinopathy (PDR) and panretinal photocoagulation for high risk PDR eyes. (XLSX)
